# Predicting the Severity of Acute Appendicitis in Children Using Neutrophil-to-Lymphocyte Ratio (NLR) and Platelet-to-Lymphocyte Ratio (PLR)

**DOI:** 10.7759/cureus.28619

**Published:** 2022-08-31

**Authors:** Adewale Ayeni, Fahad Mahmood, Ameer Mustafa, Bethan Mcleish, Vaibhavi Kulkarni, Shika Singhal, Akinfemi Akingboye

**Affiliations:** 1 General Surgery, Russells Hall Hospital, Dudley, GBR; 2 General Surgery, Walsall Manor Hospital, Walsall, GBR; 3 Surgery, The Royal Wolverhampton NHS Trust, Wolverhampton, GBR; 4 Surgery, The Dudley Group NHS Foundation Trust, Dudley, GBR; 5 Pathology, Royal Wolverhampton Hospital, Wolverhampton, GBR; 6 Colorectal Surgery, Russells Hall Hospital, Dudley, GBR

**Keywords:** children, appendectomy, inflammation, surrogate marker, biomarker, platelet-lymphocyte, neutrophil-lymphocyte, complicated appendicitis, uncomplicated appendicitis, acute appendicitis

## Abstract

Introduction

The ability to predict risk of perforation in acute appendicitis (AA) could direct timely management and reduce morbidity. Platelet-to-lymphocyte ratio (PLR) and neutrophil-to-lymphocyte ratio (NLR) are surrogate severity markers in infections. This study investigates the use of PLR and NLR as a marker for distinguishing uncomplicated (UA) and complicated appendicitis (CA) in children.

Materials and methods

This retrospective single-center study collected data between January 1, 2014, and December 31, 2020. Children between five and 17 years of age with histologically confirmed appendicitis were included. Cut-off values for NLR and PLR were determined by employing the receiver operating characteristic (ROC) curve with sensitivity and specificity in addition to regression analysis.

Results

A total of 701 patients were included with a median age of 13 years. Out of which 52% of the cohort was female. The difference between the NLR and PLR ratios between UA and CA was significant (p=0.05, Kruskal-Wallis). For UA, the area under the ROC curve (AUC) and cut-off for NLR and PLR were 0.741, 3.80 with 95% CI of 0.701-0.781 and 0.660, 149.25 with 95% CI of 0.618-0.703, respectively. In CA, using NLR and PLR, AUC and cut-off were 0.776, 8.86 with 95%CI of 0.730-0.822 and 0.694, 193.67 with 95%CI of 0.634-0.755, respectively. All were significant with p<0.001.

Conclusions

NLR and PLR are reliable, synergistic markers predicting complicated appendicitis which can guide non-operative management in children.

## Introduction

Acute appendicitis is among the most frequently encountered acute surgical presentations with a lifetime risk of 7% and the most common surgical emergency in children [1-5]. Diagnosis of pediatric acute appendicitis is based upon clinical features, although these are frequently atypical, with radiological investigations preserved for selected cases [5]. Despite the established classical symptoms and signs of acute appendicitis, prompt diagnosis of complicated appendicitis is challenging [6]. The World Society of Emergency Surgery (WSES) 2020 guidelines incorporate gangrene, perforation, and abscess in their definition of complicated appendicitis [6]. In addition, the rates of perforated appendicitis vary between 16% and 40% overall and are higher in younger age groups, ranging between 40% and 57% and are associated with diagnostic delay [7]. Thus, inability to diagnose acute appendicitis immediately on presentation may result in increased morbidity and mortality.

Numerous scoring methods to help in the quick clinical diagnosis and categorization of acute appendicitis have been developed, such as the Alvarado score, pediatric appendicitis score (PAS), the appendicitis inflammatory response (AIR), and Shera score [6,8-10]. Although each of these scores differs in their utilized clinical parameters, the overdiagnosis of appendicitis is 32% by PAS and 35% by the Alvarado score [11]. Furthermore, these scoring tools lack sensitivity and specificity in predicting the severity of acute appendicitis, although low scores can help categorize patients as low risk [6]. Thus, scoring tools are not routinely used or recommended by the WSES [6]. As a result, other parameters have been investigated to indicate the severity of appendicitis. White cell counts (WCCs), absolute neutrophil counts (ANC), and c-reactive protein (CRP) are raised in patients diagnosed with acute appendicitis [12]. Although an increase in WCC has no significant predictive value in distinguishing between uncomplicated and complicated appendicitis, WSES advises absolute neutrophil count and CRP to predict severity of inflammation [6,13,14]. Furthermore, raised blood bilirubin has been demonstrated to be a possible marker for perforated appendix but lacked sufficient sensitivity and specificity, with elevated CRP superior to bilirubin in predicting perforated appendicitis [15,16]. Identifying a marker to predict complicated appendicitis with good sensitivity and specificity can guide clinical decision-making.

Nneutrophil-to-lymphocyte ratio (NLR) and platelet-to-lymphocyte ratio (PLR) are simple inexpensive markers of inflammation, which are easily obtained [3]. Neutrophilia and lymphocytopenia are cellular response elements in systemic inflammation. The increase in the difference between neutrophil and lymphocyte reflects the severity of the inflammatory response. Therefore, NLR has been used as a marker in many pathological conditions such as malignancies, chronic inflammatory diseases, and post-operative complications for many years [17]. NLR and PLR provide information on immune and inflammatory pathways and have been studied as potential markers predicting severity of appendicitis [17]. A recent meta-analysis demonstrated that NLR predicts both diagnosis and severity of appendicitis in adults [3]. This may have implications for prioritizing cases for surgery, monitoring conservatively treated patients, and for patients who do not routinely undergo CT scans such as children. This study aimed to assess the ability of NLR and PLR to differentiate between simple and complicated appendicitis in children. We assess the sensitivity, specificity, and predictive value of NLR as a marker of severity in this age group.

## Materials and methods

Approval for the study was obtained from the Clinical Research and Audit Department of Russells Hall Hospital. A cohort study was done retrospectively in the Department of Surgery at Russells Hall Hospital, Dudley, UK, with approval number GENSUR/CA/2020-21/22.

All patients aged five to 17 years who underwent emergency appendicectomy for suspected acute appendicitis between 2014 and 2020 were identified from the hospital admission statistics (HAS) database. Included patients must have available post-operative histology, either radiological or intra-operative diagnosis of acute appendicitis and receiving either conservative or surgical management for acute appendicitis. Where patients received surgical management of acute appendicitis, we included both open and laparoscopic appendicectomy. Patients diagnosed with neoplasm of the appendix were not included in the study.

Outcomes

Our main outcome was the predictive capability of PLR/NLR in differentiating uncomplicated from complicated acute appendicitis. Secondary outcomes included evaluating the negative appendicectomy rate, presence of Enterobius vermicularis infestation, complications, and length of stay.

Data collection and analysis

From our records, two categories of patients were isolated: uncomplicated appendicitis and complicated appendicitis. A complicated appendicitis is defined as perforation of appendix anywhere from the tip to base, abscess formation, or gangrenous appendix [6]. The data gathering proforma assessed patients’ demographic data, white cell count (WBC), neutrophil count, lymphocyte count, platelet count, diagnostic modality, imaging (including ultrasound and computer tomography) findings if performed, post-operative complications using Clavien Dindo classification, duration of hospitalization, intra-operative details where operative management performed, and histology. Data were extracted separately by two members of the research team. The team members resolved any inconsistencies by engaging in dialogue. However, in the event of irreconcilable difference, a third independent member was approached.

Statistical Package for the Social Sciences (SPSS) for Windows, version 25.0 (IBM Corp.: Armonk, NY) was used to carry out the statistical analysis. The accuracy of NLR and PLR was characterized and compared using a receiver operating characteristic (ROC) curve. The area under the ROC curve (AUC) denoted accuracy in differentiating between complicated and uncomplicated appendicitis. Cut-off values were assessed for each biomarker including the sensitivity, specificity, positive predictive value (PPV), and negative predictive value (NPV) with 95% confidence intervals. A p-value of <0.05 was considered statistically significant.

Power calculation

Available literature has shown NLR of 4.7 to be a cut-off value for uncomplicated appendicitis and 8.8 for complicated appendicitis with sensitivity and specificity approaching 90% [3]. Furthermore, a cut-off for PLR of >140.45 and >163.27 for uncomplicated appendicitis and complicated appendicitis, respectively, have been documented [17]. Therefore, to ensure a study power of 80% with confidence interval (CI) of 95%, a total number of 392 patients (196 patients in each group) was the calculated minimum study size.

## Results

A total of 701 patients aged between five and 18 years of age had appendicectomies performed for suspected appendicitis between 2014 and 2020. The demographics of these patients are detailed in Table 1. The overall median age was 13±3.4 years. The gender distribution was similar with 339 males (48.4%) and 362 females (51.6%). The overall median length of stay following appendicectomy was three days (range: 1-22 days).

**Table 1:** Patient demographics - age, gender distribution, and mean and median length of stay of our patient cohort. SD: standard deviation, LOS: length of stay

Demographics	Overall	Appendix non-inflamed	Uncomplicated appendicitis	Complicated
Age mean (years)	12.98	13.68	12.65	12.79
Age median (years)	13.35	14.19	12.85	13.02
Age SD (±)	3.44	3.21	3.51	3.45
LOS (days) median	3.00	3.00	3.00	4.00
LOS (days) mean	3.26	2.83	3.13	5.04
Male	339 (48.4%)	65	228	46
Female	362 (51.6%)	145	185	32
Total	701	210	413	78

Furthermore, the majority of these patients (78.9%) had laparoscopic appendicectomies performed with an overall complication rate of 1.9% with 0.3% of these patients requiring further surgical intervention. Histology showed a 30% negative appendicectomy rate (i.e, those who had normal appendix on histology), along with a 6.1% incidence of Enterobius vermicularis. Interestingly, no patients with Enterobius vermicularis had histological evidence of appendicitis. These findings are summarized in Table 2. Biochemical markers supporting clinical decision-making including white cell count (WCC), CRP, NLR, and PLR were determined across categories of complexity (Table 3).

**Table 2:** Patient outcomes following appendicectomy. All patients with Enterobius vermicularis infestation had a histologically normal appendix.

	Percentage	Frequency
Negative appendix rate	30.0%	210/701
Enterobius vermicularis infestation	6.1%	43/701
Laparoscopic appendicectomy	78.9%	553/701
Open appendicectomy	21.1%	148/701
Complication rate	1.9%	13/701
Return to theatre	0.3%	2/701
Mortality	0.00%	0.00

**Table 3:** Biochemistry profile of the patients. The mean and standard deviation (SD) of white cell count (WCC), neutrophil-to-lymphocyte (NLR) ratio, platelet-to-lymphocyte (PLR) ratio, and c-reactive protein (CRP) levels in patients with histologically normal, uncomplicated appendicitis, and complicated appendicitis.

Variables		Mean	SD (±)
Normal appendix	WCC (x10^9^/L)	9.28	4.32
	NLR	4.48	6.45
	PLR	174.56	244.67
	CRP (mg/L)	16.76	41.71
Uncomplicated appendicitis	WCC (x10^9^/L)	12.83	4.82
	NLR	8.97	9.21
	PLR	215.14	170.52
	CRP (mg/L)	46.31	69.70
Complicated appendicitis	WCC (x10^9^/L)	16.89	5.60
	NLR	14.54	8.60
	PLR	280.06	172.13
	CRP (mg/L)	111.80	83.91

**Figure 1:**
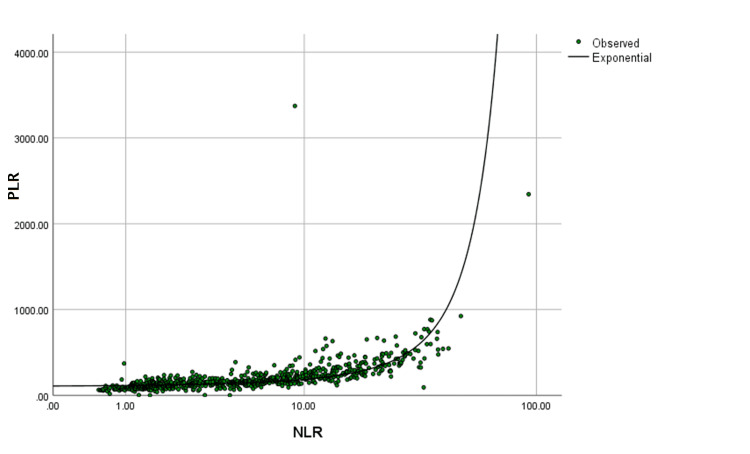
NLR and PLR exponential relationship. NLR: neutrophil-to-lymphocyte ratio; PLR: platelet-to-lymphocyte ratio

In addition, we determined the utility of the neutrophil-to-lymphocyte ratio (NLR) and platelet-to-lymphocyte ratio (PLR) in predicting uncomplicated versus complicated appendicitis. A curve estimation showed that NLR and PLR exhibit an exponential relationship (R^2^=0.478, p<0.001) (Figure 1).

**Figure 2:**
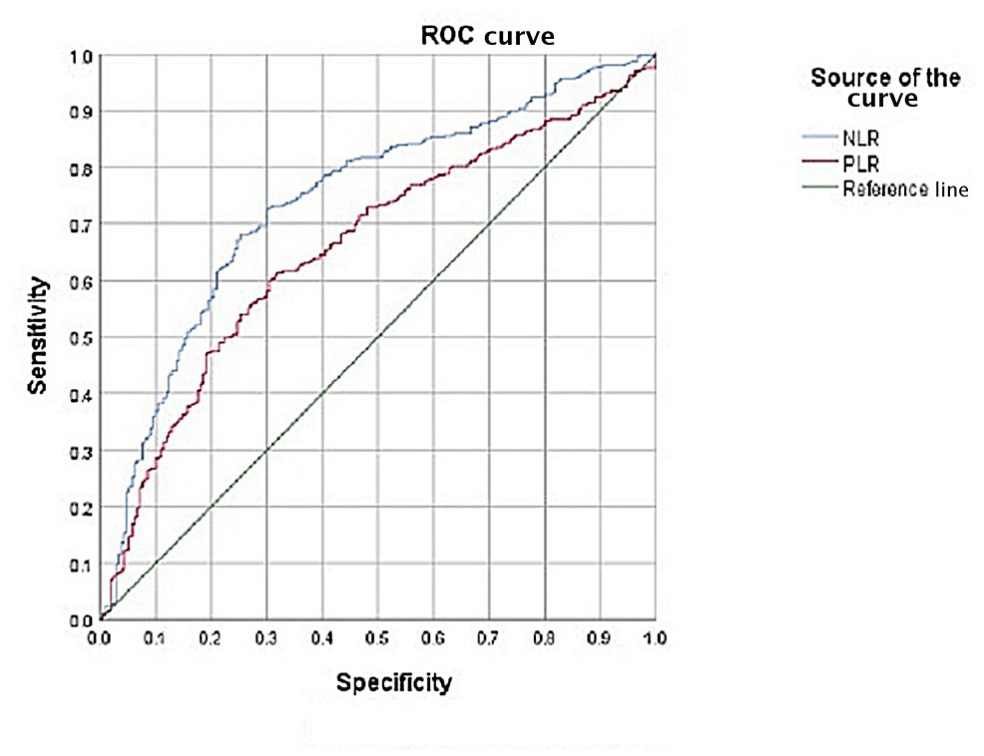
NLR and PLR ROC curve for uncomplicated appendicitis. Diagonal segments are produced by ties. NLR: neutrophil-to-lymphocyte ratio; PLR: platelet-to-lymphocyte ratio; ROC: receiver operating characteristic

**Figure 3:**
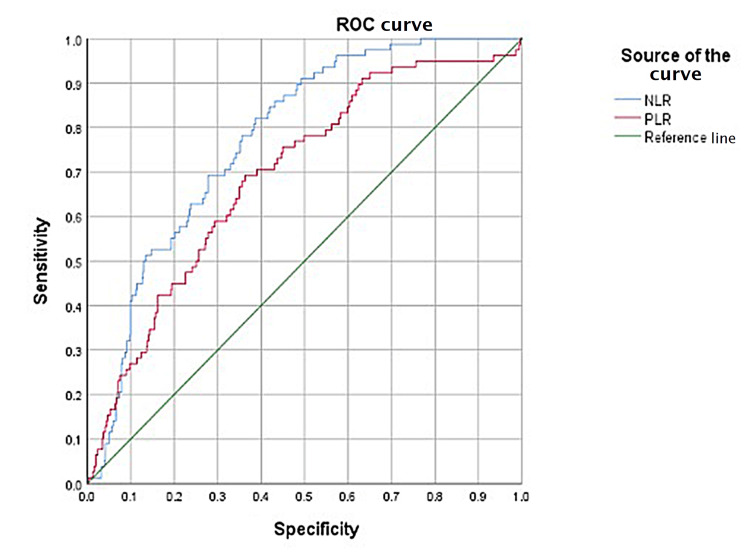
NLR and PLR ROC curve for complicated appendicitis. NLR: neutrophil-to-lymphocyte ratio; PLR: platelet-to-lymphocyte ratio; ROC: receiver operating characteristic

A Kruskal-Wallis test showed a statistically significant difference between complicated and uncomplicated appendicitis for both NLR and PLR (p<0.001). ROC curves for NLR and PLR both had statistically significant area under the ROC curve (AUC) differentiating complicated and uncomplicated appendicitis (p<0.001) (Figures 2, 3). The cut-offs for uncomplicated appendicitis (NLR=3.80, PLR=149.25) and complicated appendicitis (NLR=8.86, PLR=193.67) were calculated from the ROC curves and used to measure inter-rater agreement between NLR and PLR.

Cohens’ and Fleiss’ kappa were used to calculate agreement between NLR and PLR for uncomplicated and complicated appendicitis, respectively (Table 4). This showed weak-moderate agreement for uncomplicated appendicitis (0.574, p<0.001) and moderate agreement for complicated appendicitis (0.530, p<0.001). Furthermore, cross-tabulation of NLR, PLR as well as CRP for sensitivity, specificity, positive predictive value, and negative predictive value using the above cut-off values in determining severity of appendicitis are shown below in Table 5.

**Table 4:** Cohen and Fleiss symmetric measures. *Not assuming the null hypothesis. **Using the asymptotic standard error assuming the null hypothesis.

Cohen	Value	Asymptotic standard error*	Approximate**	Approximate significance
Measure of agreement kappa	0.574	0.031	15.217	0.000
Number of valid cases	701	-	-	-
Fleiss				
Measure of agreement Kappa	0.530	0.026	19.654	0.000
Number of valid cases	701	-	-	-

**Table 5:** Sensitivity, specificity, positive predictive value, and negative predictive value for differentiating uncomplicated and complicated appendicitis. NLR: neutrophil-to-lymphocyte ratio; PLR: platelet-to-lymphocyte ratio; CRP: c-reactive protein

	Sensitivity % (95% confidence interval)	Specificity % (95% confidence interval)	Positive predictive value % (95% confidence interval)	Negative predictive value (95% confidence interval)
NLR	70.3 (66.2-74.3)	70 (63.8-73.1)	84.6 (81.1-88.1)	50.2 (44.4-55.9)
PLR	64.0 (59.7-68.2)	61.0 (54.4-67.6)	79.3 (75.3-83.3)	42.0 (36.4-47.5)
CRP	70 (65.9-74.1)	71.8 (65.6-78.0)	85.2 (81.6-88.7)	50.9 (45.1-56.7)

## Discussion

Our results show concordance between NLR and PLR in discriminating between uncomplicated appendicitis and complicated appendicitis in children. Based on our analysis, NLR has a sensitivity of 70.3% and a specificity of 70% with a positive predictive value (PPV) of 84.6% and a negative predictive value (NPV) of 50.2% (Table 4). Moreover, PLR has a sensitivity of 64% and a specificity of 61% with a PPV of 79.3% and a NPV of 42%. This was similar to the more established marker CRP, which has a sensitivity of 70%, specificity of 71.8%, PPV of 85.2%, and NPV of 50.9%. According to McGowan et al., CRP had a sensitivity of 46-94% and a specificity of 32-84% considering the cut-off used which ranged from >5 to >100 [18]. The PPV of CRP ranges from 16% to 25% but has an NPV of 91-97%. The PPV of all three parameters was most discriminating when predicting severity of appendicitis in the pediatric population, i.e., patients with a CRP>111 mg/L, an NLR>14, and a PLR>280 were more likely to have complicated appendicitis. In addition, the PLR and NLR showed concordance in their tendency to predict severity of appendicitis in children as shown statistically by the inter-rater agreement analysis. Our study shows significant correlation between PLR and NLR allowing for a moderate degree of agreement between simple and complicated appendicitis in pediatric patients. This demonstrates that when NLR and PLR are considered together, the reliability of diagnosing both complicated and uncomplicated appendicitis increases significantly. Thus, NLR and PLR can be used synergistically to identify more severe cases of acute appendicitis and serve as valuable tools in clinical decision-making for pediatric appendicitis.

These markers (NLR and PLR) can both be easily calculated from the complete blood count (CBC) differential, thus are inexpensive markers. This has practical implications for ease of use and avoids reliance on more complex scoring systems which usually require reference to online calculators or text-based scoring systems [6,8,9]. However, the sensitivity and its specificity can differ significantly depending on the time the test was conducted. Tests carried out within hours of the onset of symptoms may be normal, only to increase in subsequent hours [6,19]. Considering the variability in the timing of presentation of patients to secondary care with respect to their onset of symptoms, it is paramount to interpret these tests in context of the clinical presentation. While we recognize this limitation in our study, we believe that a pragmatic approach to interpretation of results is a vital part of medical practice and further stratifying patients based on their onset of symptoms, which in itself can be extremely unreliable, would limit the practical applicability of NLR and PLR in children with acute appendicitis.

Our study provides further validation of previously published data evaluating the utility of NLR and PLR in predicting appendicitis severity. In the meta-analysis by Hajibandeh et al., NLR >4.7 and >8.8 were independent predictors of uncomplicated appendicitis and complicated appendicitis, respectively [3]. This corresponds with our NLR cut-off value of 8.86 for complicated appendicitis. In addition, Pehlivanlı and Aydin reported that a PLR> 140.45 has a sensitivity of 71.4% and specificity of 88.9% to distinguish between appendicitis and a normal appendix, whereas a PLR >163.27 has a sensitivity of 64.3% and a specificity of 67.5% to differentiate complicated appendicitis from uncomplicated appendicitis [17]. However, our data suggest a much higher cut-off value of >193.67 with a sensitivity of 64% and a specificity of 61%. The level and unit of measurement of PLR in the literature vary due to heterogeneity between study populations in addition to geographical and ethnic differences. In a recent meta-analysis, Liu et al. used standardized mean difference to account for this and demonstrated significant increase in PLR level in simple appendicitis compared to controls (standardized mean difference {SMD}: 1.23, 95% CI: 0.88-1.59) but was unable to demonstrate this effect when differentiating between simple appendicitis and complicated appendicitis (SMD: 2.28, 95% CI: -1.72-6.28) [20]. Thus, although raised PLR may indicate severity of appendicitis, this parameter should be interpreted with caution in isolation. These results also argue for the development of more nuanced clinical scoring systems that have a greater sensitivity and specificity than established scores that have not gained widespread use. In the setting of a busy emergency surgical on-call, the NLR and PLR can help in the prioritization of patients for surgery. Moreover, there has been an increasing trend toward non-operative management of patients [21-23]. The risk of recurrent appendicitis requiring operative intervention in adult patients managed non-operatively is 30% in 90 days [23], whereas up to 25% of children require appendicectomy at one year with a higher risk of complicated appendicitis [22,24]. Nonetheless, non-operative management can be considered in selected cases where complicated appendicitis is not expected. Conversely, in cases of operative management, appropriate stratification for cases suited to training can be based on pre-operative risk stratification to determine complexity.

Finally, negative appendicectomies remain a major problem, particularly in practice in the United Kingdom (UK). In our study, the negative appendicectomy rate in children was relatively high at 30% which is higher than the 15.9% reported in the UK national RIFT audit [9]. Similar to the findings of the right iliac fossa pain treatment (RIFT) audit, young females with concomitant gynecological pathologies were found to be responsible for the majority of negative appendectomies carried out. In our practice, if no other significant explanatory pathology is found during a diagnostic laparoscopy, we advise removal of a macroscopically normal appendix. This practice may be further justified by the high incidence of Enterobius vermicularis found in children which may explain their presentation. Although, increasingly CT scan is being used in investigating acute right iliac fossa pain and diagnosing acute appendicitis in adults [9,25,26]. While this remains routine practice in many countries, its use has been limited in the UK with greater reliance on clinical diagnosis. Furthermore, the ionizing radiation risk of CT scans is difficult to justify for children and pregnant women. Despite the low morbidity associated with negative appendicectomy, NLR and PLR could be used in the future to identify children with acute appendicitis prior to surgery where a CT scan cannot be performed.

Our study being a single-center retrospective cohort study makes it impossible to eliminate entirely the possibility of confounding factors including the time of testing and machine calibration. Despite the nuances of UK practice, our findings in pediatric practice may have widespread application. A further multicentre prospective study would also increase the applicability and validate these scoring measures.

## Conclusions

NLR and PLR ratios are propitious markers that can predict both diagnosis and severity (uncomplicated vs complicated) of appendicitis in children and when interpreted together could have acceptable sensitivity and specificity. Our data support the use of NLR and PLR to risk stratify children with either clinically confirmed appendicitis in resource-constrained environments with limited theatre facilities or where repeat imaging is not readily available. It can also be used to monitor pediatric patients with appendicitis who are being treated non-operatively or support the diagnosis in patients where CT imaging is not justified such as children. Further studies are required to assess whether combining NLR and PLR with other markers such as CRP would result in better predictive value.
